# Glomangiosarcoma Involving the Heart with an Unknown Primary Lesion

**DOI:** 10.7759/cureus.2907

**Published:** 2018-07-01

**Authors:** Komal Ejaz, Muhammad A Raza, Abdul Aleem, Shahram Maroof, Hassan Tahir

**Affiliations:** 1 Medicine/Sheikh Zayed Hospital, University of Health Sciences, Rahim Yar Khan, PAK; 2 Internal Medicine, Jinnah Hospital Lahore/allama Iqbal Medical College, Lahore, PAK; 3 Internal Medicine, St. Mary Mercy Hospital, Livonia, USA; 4 Pulmonary Critical Care, John H. Stroger, Jr. Hospital of Cook County, Chicago, USA; 5 Cardiology, USA Medical Center, Mobile, USA

**Keywords:** glomangiosarcoma, glomangiosarcoma involving the heart, glomus tumor, extracutaneous glomus tumor, rare heart tumors

## Abstract

Glomus tumors are usually benign tumors of the glomus cells with the immunocytochemical and structural features of smooth muscle cells. The majority of the cases of glomus tumors are benign but, rarely, they demonstrate malignant features both clinically and histologically (also known as glomangiosarcomas). Although glomangiosarcoma involving extracutaneous sites is uncommon, a few cases have been reported. A glomangiosarcoma of the heart is extremely rare due to the rarity of glomus bodies in the myocardium. In this case report, we present the case of a 31-year-old female with glomangiosarcoma involving the heart with an unknown primary lesion.

## Introduction

Glomangiosarcoma usually arises from a primary glomus tumor. These tumors commonly involve the skin, where an abundance of glomus bodies is present for temperature regulation in the dermis [1]. An exhaustive literature review on glomangiosarcoma failed to reveal any previous cases of myocardial involvement. To the best of our knowledge, this is the first case report on glomangiosarcoma involving the heart.

## Case presentation

A 31-year-old Caucasian female presented with progressive dyspnea and palpitations for three months. The patient reported gradual onset, intermittent dyspnea aggravated with walking, relieved with rest, and associated with palpitations. Her past medical, surgical, and family history were non-contributory. The patient denied fever, cough, chest pain, orthopnea, diaphoresis, excessive caffeine intake, or smoking/alcohol/illicit drug use. The relevant findings on physical examination included a pulse rate of 96/minutes and a blood pressure of 135/89 mmHg. On cardiac auscultation, a diastolic murmur of grade 3/6 over the left fifth intercostal space in the midclavicular line was appreciated. The electrocardiogram (ECG) showed atrial flutter at the rate of 122 beats per minute with a rapid ventricular response.

Laboratory investigations, including complete blood cell count, electrolytes, thyroid profile, and liver enzymes were within the normal range. The erythrocyte sedimentation rate (ESR) was 56 mm/hr, c-reactive protein (CRP) was 12.6 mg/dl, lactate dehydrogenase was 587 U/l, and the N-terminal-pro-B-type natriuretic peptide was 1654 pg/ml.

**Figure 1:**
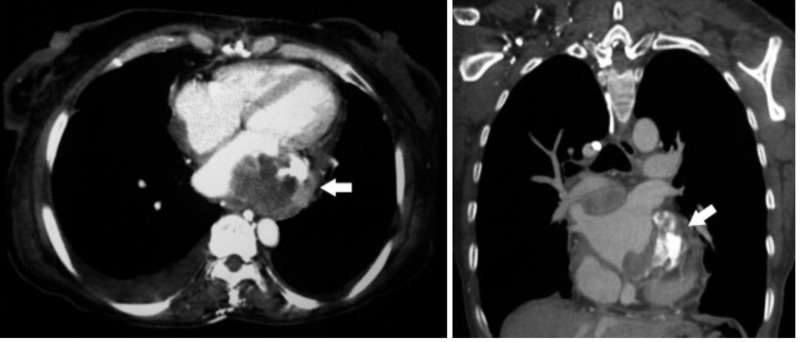
Thoracic computed tomography (CT) showing an irregular left atrial mass (arrow) attached to the posterior wall

Transthoracic echocardiography revealed an irregular heterogeneous mass in the left atrium, adhering to the posterior leaflet of the mitral valve, leading to mild-moderate mitral stenosis. Cardiac computed tomography (CT) confirmed the presence of an irregular solid mass attached to the posterior wall of the left atrium (Figure 1). Systemic CT scans did not reveal any other masses. A diagnosis of atrial myxoma was considered and surgical resection was planned. Intraoperative examination revealed a mass adherent to the posterior wall of the left atrium and the posterior mitral leaflet. Complete resection could not be performed due to the extension of the tumor into adjacent structures. Surgery was well-tolerated and the patient was shifted to the floor.

**Figure 2:**
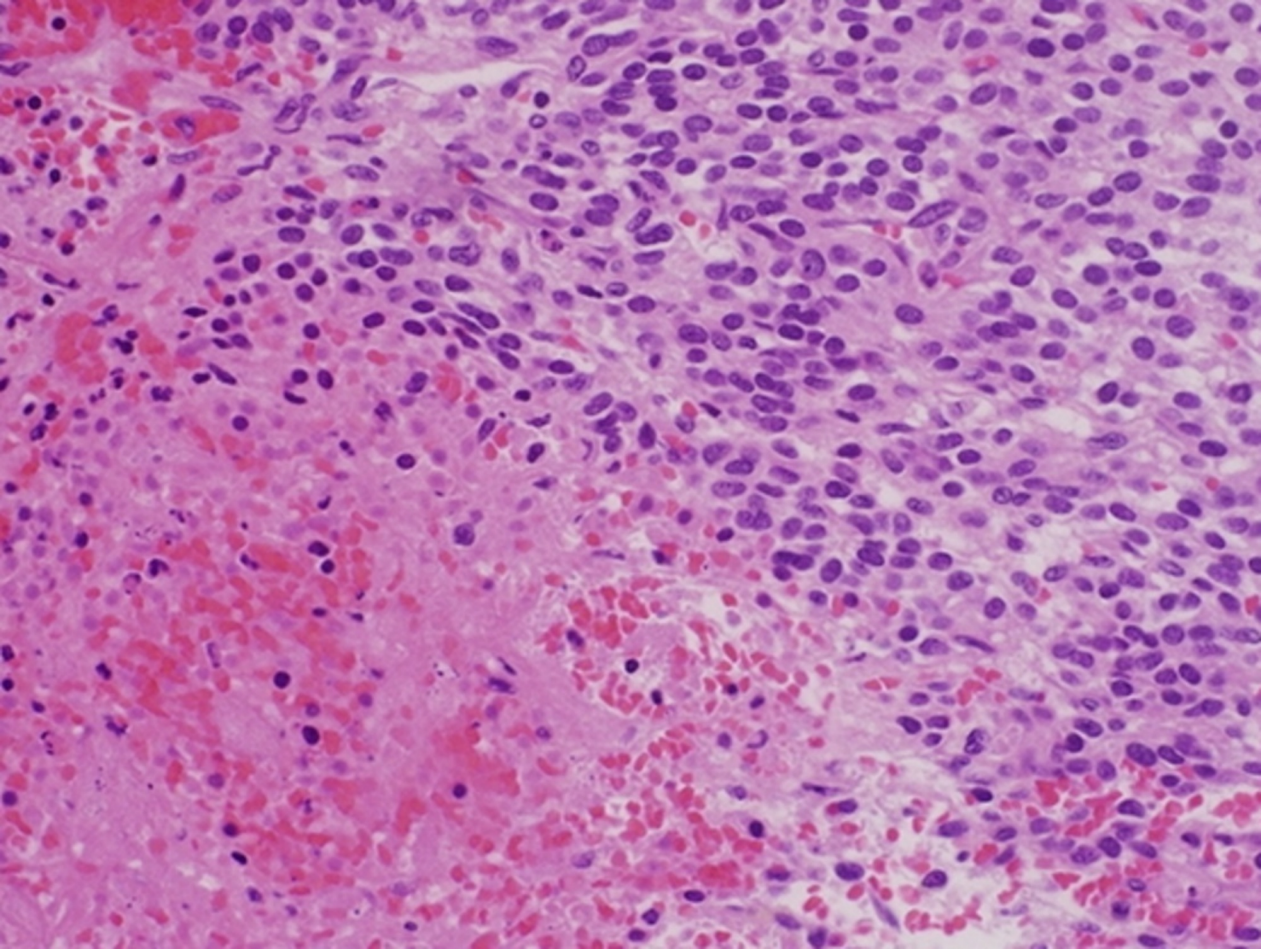
Histopathology of the excised cardiac tumor showing polygonal cells with prominent nuclei and surrounding vascular structures

Macroscopically, an irregular solid mass of 2.1×1.7×1.6 cm was found. The non-encapsulated tumor consisted of highly vascularized beige-to-pink tissue. Microscopically, the tumor was composed of polygonal cells with centrally placed round to oval nuclei and a prominent nucleolus. The cells were surrounded by thin-walled capillary-sized vascular structures and areas of coagulative necrosis. Mitotic activity was approximately 7-8/50 high-power field (Figure 2). Immunohistochemically, the tumor cells were positive for vimentin and actin but were negative for desmin, CD34, and S-100. The final pathological diagnosis was de-novo intracardiac glomangiosarcoma. The patient recovered uneventfully and was discharged home in a stable condition. A follow-up visit after three months revealed normal radiological examination findings.

## Discussion

Glomus tumors and glomangiosarcomas most frequently involve the dermis and subcutaneous tissues because of the predominance of glomus bodies in these sites. Glomus bodies are involved in temperature regulation and have vascular components surrounded by multiple layers of epithelioid cells and nerve fibers. Extracutaneous glomangiosarcomas are exceedingly rare due to the deficiency or near absence of glomus bodies in sites other than the skin. Nevertheless, glomangiosarcomas have been reported in deep visceral organs, such as the gastrointestinal tract, liver, mediastinum, and respiratory tract [2], uterus, vagina [3], and brain. To the best of our knowledge, this is the first case report of a glomangiosarcoma involving the heart.

Glomangiosarcomas mostly present as a solitary lesion, but multiple lesions can also be seen. Solitary tumors are more common in adults while multiple lesions are usually seen in children. One of the case series on glomangiosarcomas highlighted a metastatic rate of 38% among all malignant cases [4]. Glomangiosarcomas can be subdivided into two categories on the basis of their origin, which can be de-novo or from a benign glomus tumor. The latter has a malignant component mixed with a typical glomus tumor. Histologically, benign glomus tumors consist of solid nests and sheets of round to polygonal cells interrupted by capillary-sized vessels. The malignant counterpart shows atypia and high mitotic index and has increased expression of P53 and B-cell lymphoma  (BCL-2), which is not shown by benign glomus tumor cells [5].

On the contrary, de novo glomangiosarcoma is more difficult to diagnose because of the absence of benign glomus tumor histology and can be easily confused with other sarcomas. Immunohistochemical staining is helpful in the diagnosis of glomangiosarcoma, as tumor cells are typically positive for smooth muscle actin and vimentin but negative for epithelial, vascular, and neural markers (i.e. desmin, cytokeratin, S100, chromogranin, and neurofilament) [2,6].

Imaging studies can also be helpful in the localization and diagnosis of glomangiosarcomas of deep visceral organs, especially the heart. A computed tomography (CT) scan usually shows an irregular solid mass that can invade surrounding tissue. An echocardiogram (EKG) shows a heterogeneous mass. Hypervascularity is a typical feature of glomangiosarcoma leading to contrast enhancement on myocardial perfusion imaging.

The most effective treatment of glomangiosarcomas is wide surgical excision with good prognosis. There is no recommendation for the use of radiotherapy or chemotherapy even in the presence of metastasis. However, the local recurrence rate of glomangiosarcomas is high, so long-term follow-up is generally recommended [2].

## Conclusions

Glomangiosarcomas are often misdiagnosed as other soft tissue tumors. Despite being rare, glomangiosarcomas should be considered in the differential diagnosis of extracutaneous tumors. Immunohistochemistry and pathology are essential in diagnosing glomangiosarcomas and should be performed in all suspicious cases of soft tissue tumors. Glomangiosarcomas metastasize frequently and have a high local recurrence rate, so a vigilant follow-up is recommended even after complete resection.
